# The Influence of Stress on Decision-Making: Effects of CRF and Dopamine Antagonism in the Nucleus Accumbens

**DOI:** 10.3389/fpsyt.2021.814218

**Published:** 2022-01-25

**Authors:** Rapheal G. Williams, Kevin H. Li, Paul E. M. Phillips

**Affiliations:** ^1^Center for Excellence in Neurobiology of Addiction, Pain and Emotion, Seattle, WA, United States; ^2^Graduate Program in Neuroscience, Seattle, WA, United States; ^3^Department of Psychiatry and Behavioral Sciences, Seattle, WA, United States; ^4^Department of Pharmacology, University of Washington, Seattle, WA, United States

**Keywords:** dopamine, CRF, stress, nucleus accumbens, decision making

## Abstract

The actions of corticotropin-releasing factor (CRF) in the core of the nucleus accumbens including increasing dopamine release and inducing conditioned place preference in stress-naïve animals. However, following two-day, repeated forced swim stress (rFSS), neither of these effects are present, indicating a stress-sensitive interaction between CRF and dopamine. To ascertain the degree to which this mechanism influences integrated, reward-based decision making, we used an operant concurrent-choice task where mice could choose between two liquid receptacles containing a sucrose solution or water delivery. Following initial training, either a CRF or dopamine antagonist, α-helical CRF (9–41) and flupenthixol, respectively, or vehicle was administered intracranially to the nucleus accumbens core. Next, the animals underwent rFSS, were reintroduced to the task, and were retested. Prior to stress, mice exhibited a significant preference for sucrose over water and made more total nose pokes into the sucrose receptacle than the water receptacle throughout the session. There were no observed sex differences. Stress did not robustly affect preference metrics but did increase the number of trial omissions compared to their stress-naïve, time-matched counterparts. Interestingly, flupenthixol administration did not affect sucrose choice but increased their nosepoke preference during the inter-trial interval, increased trial omissions, and decreased the total nosepokes during the ITI. In contrast, microinjections of α-helical CRF (9–41) did not affect omissions or ITI nosepokes but produced interactions with stress on choice metrics. These data indicate that dopamine and CRF both interact with stress to impact performance in the task but influence different behavioral aspects.

## Introduction

Perturbations in the dopamine monoaminergic system contribute to several psychiatric disorders, including Major Depressive Disorder (MDD). The dopamine-rich nucleus accumbens (NAc) serves as an integrator of limbic, motor, and cognitive information and may act as a hub between stress and reward-responses, leading to affective action selection (1, 2). The induction of chronic stress and trauma is a significant factor expediting the onset of Major Depressive Disorder (MDD) where a small motivational stressor, such as making a new friend or deciding between assorted brands of food, becomes a large obstacle for optimal performance (3).

Corticotropin-releasing factor (CRF) is released in the paraventricular nucleus of the hypothalamus in response to stress and, more generally, when an individual experiences invigorating stimuli, regardless of the affective value (4–6). However, CRF and its two receptor subtypes, CRFR1 and CRFR2, are not only found in the anterior pituitary, but are also distributed throughout the brain, including dopamine-rich areas such as the ventral tegmental area (VTA), NAc and prefrontal cortices (7, 8). Accordingly, there are interactions between CRF and dopamine in these areas [e.g., (9–13)]. Within the NAc, local injections of CRF can augment both cue-elicited sucrose-seeking (14) and partner preference (15), behaviors that have been linked to dopamine transmission (16, 17). Work from our lab has shown directly that CRF administration potentiates dopamine in the NAc and produces a positive affective state, but this potentiation is abolished following exposure to repeated forced swim stress (rFSS) with a concomitant shift in affective response to CRF to aversion (12).

In more complex decision-making behaviors, CRF and stress increase choice latencies, reduce progressive-ratio breakpoints to choose, and reduce rates of lever pressing (13, 18, 19). In the current work, we sought to test whether CRF's actions in the NAc on decision making can be directly attributed to its regulation of mesolimbic dopamine, and how this relationship is disrupted following stressor exposure. Using a concurrent-choice task, we investigated affective decision-making in male and female mice. We hypothesized that CRF antagonist effects should be qualitatively similar to those of dopamine antagonists in stress-naïve animals, but following rFSS, overall behavioral vigor in the task would be reduced and the effects of CRF and dopamine antagonism would be disassociated. Surprisingly, we found minimal effects of stress alone on this task. However, stressor exposure changed the effects of CRF and dopamine antagonists. Contrary to our working hypothesis, CRF antagonists primarily modified the preference between the concurrently available rewards, but dopamine antagonists modified engagement in the task without affecting choice. These data suggest that both CRF and dopamine influence decision making, but they do so in parallel influencing different aspects of the task.

## Methodology

### Subjects

All animal procedures were performed in accordance with the University of Washington Institutional Animal Care and Use Committee. Eighty-eight C57/Bl6 mice (46 male and 42 female) in multiple cohorts were used for all experiments and weighed between 17 and 25 g prior to food restriction and training. All mice were single-housed in a temperature- and humidity-controlled vivarium on a 12L: 12D cycle (lights on at 07:00) and food restricted above 85% of free-feeding body weight after at least 1 week of acclimation and surgery recovery. Animals who did not receive cannulation surgery (*n* = 20) were food restricted 1 week after acclimation to being housed individually. Water was provided *ad libitum*. Animals were weighed before each training or testing session and provided with standard rodent chow after the session.

### Apparatus

Testing was conducted in Plexiglas operant chambers (24 × 20 × 21 cm; ENV-307W; Med Associates, St. Alban, VT, USA) that were contained within in-house built, sound attenuating cubicles. Each chamber contained two liquid receptacles (ENV-300R1AM) on the right and left sides, 4 cm above the grid floor, that were equipped with infrared sensing, head entry detectors (ENV-303HDW). These were used for animals to nosepoke for either water or a 0.1 M sucrose solution. The liquid delivery was controlled by a solenoid opening (100 ms) and pressurized air to deliver 5 μL of liquid for each reinforced choice.

Either a flashing (1 Hz) or a continuous, LED stimulus light (ENV-321W) was positioned over each receptacle 8 cm above the grid floor. A mounted speaker and audio generator (ENV-223) positioned on the rear wall of the chamber was used to signal the availability of either liquid with either 8- or 15-kHz noise for forced-choice trials. Free-choice trials where both liquids are available were signaled by illumination of both visual cues and 4-kHz noise. The liquid reinforcer associated with visual and auditory cues was counterbalanced across animals. These behavioral protocols were controlled by Med-PC software and the data was stored for offline analysis. Video was recorded using an infrared-sensitive camera and DVR system (Zosi ZG2111C) and analyzed using Ethovision XT video analysis software (Noldus Information Technology).

### Nosepoke Training

Animals underwent magazine training, and fixed-ratio one training, side bias assessment, and sucrose preference training, before eight testing sessions on the task itself. Following at least 5 days of food restriction, animals were introduced to sucrose (10 sucrose pellets) in their home cage and began operant training to following day. First, “magazine training” exposed the animal to the discrete cues associated with either the left or right liquid receptacle by continuous visual and auditory stimulus presentation with free deliveries of sucrose solution over 90 min. On reward delivery, the stimulus was terminated and then resumed 1-s later. During these sessions, additional liquid could be earned by emitting a nose-poke response into the receptacle. Behavior was further shaped for the first 2 days by adding half a crushed sucrose pellet into the receptacle. The number of free rewards during magazine training was 48 for the first two sessions, 24 on the third session and 12 thereafter. Therefore, after the first two sessions, animals were required to make nose-poke responses to receive the maximum 48 rewards. Once animals earned 40 rewards in a magazine training session they transition into fixed-ratio one (FR1) training. The FR1 training consisted of alternating, pseudorandom forced-choice trials which started with discrete cue (visual and auditory) presentation for either the left or right side until the animal successfully nosepoked into the receptacle to retrieve the reward (0.1 s, 0.1 M sucrose delivery) or trial timed out. These training days took seven to ten sessions for all animals to complete at least 24 trials with optimal performance of completing 40–48 trials. Over the sessions the time allowed to initiate the reward delivery before going to the next trial went from an indefinite time to 40 s (∞, 120 s, 60 s, 40 s) and the inter-trial interval (ITI) increased from 5 ± 2 s to 40 ± 10 s (5 ± 2 s, 10 ± 5 s, 20 ± 5 s, 30 ± 10 s, 40 ± 10 s). Sessions were completed when the animal reached 48 reinforced trials or after 90 min. Once animals reached criterion, their inherent side preference was tested by replacing 24 of the forced-choice trials with free-choice trials. Animals were then assigned sucrose solution to either the left or right side for the remainder of the experiment in a counterbalanced fashion. That is, half of the animals were assigned sucrose in receptacle on their preferred side and the other half assigned water on their preferred side.

### Concurrent Choice Decision-Making Task

**Figure 1:**
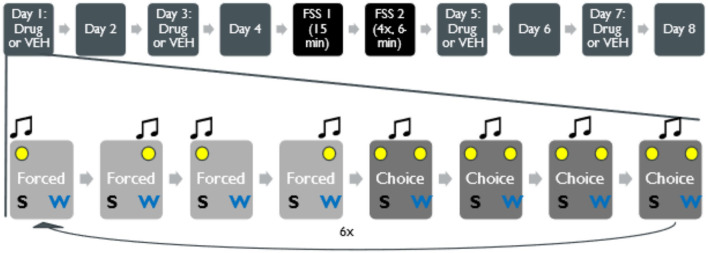
Structure of concurrent-choice operant conditioning task in a within-subjects design. Top, following conditioning, mice underwent 8 days of decision-making task: stress naïve behavior was recorded for 4 days before rFSS (or no stress) and 4 days after the stress. On days 1, 3, 5, and 7, in a counterbalanced fashion, animals designated to receive drug microinjection were injected with drug into the NAc via bilateral cannula implantation 15 min before the start of the task. The rFSS was conducted over 2 days. The first day, animals swam for 15 min, and the second day, animals swam 4 times for 6 min each time. Animals in a no stress condition could rest for 2 days. Bottom, within a single session, there were 48 trials, half of which were forced choice trials organized in a pseudorandom order. Following 4 trials of forced-choice for either water or sucrose with audio (8 or 15 kHz) and visual cues (cue light directly above the associated receptacle), 4 free-choice sessions were available (4-kHz noise and both cue lights on). This was repeated 6 times throughout the session for a total of 48 trials.

Following magazine and FR1 training on sucrose solution, animals then were introduced to 60-min sessions where the animal could choose between sucrose and water. The session consisted of 48 trials separated into six blocks of eight trials that began with pseudorandom presentation of four forced-choice trials (visual and 8 or 15 kHz auditory cues for left or right receptacle availability) and four free-choice trials (visual cues on both sides and 4-kHz noise). Mice had 40 s to respond to either side before the cues turned off and a 40 ± 10 s ITI commenced. Animals took five to seven sessions to reach 75% preference for the sucrose receptacle in free-choice trials. After reaching criterion, there were four testing sessions on the same task, followed by the 2-day repeated forced swim stress (rFSS), and then four more testing sessions. For this within-subjects design mice receiving intra-accumbal microinjections were infused on test sessions one and three with no infusions on sessions two and four to allow the drug washout. Following rFSS, mice received infusions on test sessions five and seven, in the same fashion, with no infusions on sessions six and eight. Mice under stress-naïve conditions (*n* = 13) performed the task on the same schedule, but with 2-day break between test sessions four and five (time matched to rFSS), to control for time-dependent effects of the behavior and/or repeated drug exposure (Figure 1).

### Repeated Forced Swim Stress

To induce chronic stress, we exposed mice to a 2-day, repeated forced swim stress. This was a modified Porsolt forced-swim paradigm described in by McLaughlin et al. (20, 21). Mice swam in 30°C water for 15 min on the first day, but the second day included four 6-min swims to increase immobility times for every swim. There were no opportunities for the animals to escape by making sure the water was 10 cm from the rim of the bucket and high enough for the tails to never reach the bottom of the 5-L bucket. During the stress, we measured immobility times for all animals to observe if there were any effects of sex, cannulation surgery, or drug pre-exposure. All animals were sufficiently dry before returning to their home cages.

### Cannulation Surgery

Mice were fully anesthetized with isoflurane gas in an induction chamber with 5% isoflurane for 2 min before their shaving and injection with analgesic (Meloxicam, 5 mg/kg s.c. in 1 mL of saline). Weights were taken before anesthesia induction. Animals were then secured in a stereotaxic frame with ear and bite bars to maintain a stable and flat skull surface. For the duration of the aseptic surgery animals were kept on a 1.5–2.0% isoflurane in oxygen gas for a reliable surgical plane. Bilateral cannulae were then implanted to 1 mm above the nucleus accumbens core [NAcc; coordinates, anteroposterior (AP) +1.2 mm from bregma, mediolateral (ML) +1 mm from bregma, and dorsoventral (DV) −3.5 mm from the skull surface]. Double-guide cannulas (26 gauge, 3.5 mm from the pedestal, 2-mm separation; Plastics One) were anchored using a skull screw and dental acrylic cement. Dummy internal cannulas were kept inside the cannula until injection using a 33-gauge internal cannula (Plastics One). For post-operative care, analgesic (Meloxicam, 5 mg/kg dissolved in 1 mL of saline) was administered 24 h after the surgery and weights were taken daily. Animals were allowed 7 days of recovery before initial behavioral training.

### Drug and Microinjection Protocol

Before the first injection, animals were habituated to handling and the microinfusion procedure via insertion of internal cannula and allowing the animal to roam in a separate, clean Plexiglas homecage following their sucrose preference training session without infusing any liquid to emulate the time (~5 min.) and handling it requires for drug administration. In this within-subjects design, animals were infused with drug or vehicle, counterbalanced, on test session one and three, and five and seven, (i.e., two infusions before and two following stress-induction). For example, if an animal were injected with a drug on test session one, they would be injected with a vehicle on session three. After the 2-day rFSS, they would be injected with drug on test session five and vehicle on session seven. The other half of the animals received vehicle on test sessions one and five and drug on sessions three and seven.

We used a non-selective CRF antagonist, α-helical CRF_(9−41)_ (500 ng/200 nL; Tocris Bioscience; *n* = 17) or its vehicle (0.01% acetic acid in lactated ringer's solution; *n* = 17), to test its effects on decision-making. We chose the 500 ng/200 nL since our lab has shown that they can reduce stress-induced changes in novel object exploration (in stress-naïve mice) and progressive ratio breakpoints (12, 13). Regarding dopamine antagonism, we locally infused flupenthixol (20 μg/0.5 μL; *n* = 19), to see what components of our behavior would be influenced compared to its vehicle (physiological saline; *n* = 19). This dose was chosen since it was shown to decrease sign-tracking behaviors, reduce lever pressing probability, and increase lever-pressing latency in rats (22, 23). It has also been shown to decrease rates of food-operant responding when injected into the NAc (24). This dose was also the only effective dose used in a disconnection experiment to influence ethanol CPP, although it was unilaterally injected into the amygdala (25). All solutions were microinjected at a rate of 125 nL min^−1^, and the internal cannula was left in for an additional minute to diffuse into the tissue. Mice were then left in their homecages for 15 min prior to their session.

### Histology

Mice were deeply anesthetized with a ketamine/xylazine cocktail (ketamine 75.8 mg/mL, xylazine 4.8 mg/mL) at a volume of 0.1 mL/20 g for a total of 7.58 mg ketamine before intracardial perfusion. We microinfused Chicago SkyBlue to verify injection sites before brain removal, fixation in 4% paraformaldehyde, and preservation in 30-% sucrose solution in phosphate-buffered saline. Brains were sectioned at 40 μm, mounted on slides. Animals without correct placement or had surgical complications were not included in histology or data analysis (*n* = 15). Figure (see in Supplementary Figure 1) represents the ventral portion of the injection sites in the NAc. All cannula traces and dye stains were demarcated in a blind fashion.

### Data Analysis

To verify that the animals were following the cues in a manner that reflected more than just exploratory behavior and baseline responding, we compared the average free- and forced-choice latencies to 50% of the ITI response interval. To calculate the response interval, we multiplied 48 trials by the 40 s average ITI then divided that number (1920 s) by the total amount of nosepokes an animal made during the session to get the average interval of nosepokes made during the ITI (NPI). A two-way ANOVA was used comparing the session day and the time calculated from each metric.

To assess performance during testing, we measured reinforced choice (percentage of completed free-choice trials where mice selected sucrose), omissions (number of trials where mice did not respond during cue presentation), baseline responding (number of responses during intertrial intervals), and baseline preference (percentage of total responses during intertrial intervals that were in the sucrose receptacle). These metrics were grouped into preference (reinforced choice and baseline preference) and engagement (omissions and baseline responding). Analyses of response latencies did not prove to be especially informative but are included in the supplementary figures for transparency. We also measured and analyzed immobility times during the rFSS across cohorts (see in Supplementary Figure 6). Sex-differences between male and female mice were tested for the aforementioned dependent variables.

The concurrent-choice data was analyzed using two-way, repeated measures ANOVA, with drug treatment and stress-state (pre-rFSS and post-rFSS) as two within-subject factors. For animals without surgery, factors were stress treatment and stress-state in individual days (i.e., 1–4 vs. 5–8). To compare sex-differences within the task three-way, repeated measures ANOVA was used with sex as the third factor, but a final comparison of males and females in a stress-naïve condition were compared using an unpaired *t*-test. Mixed-effects analysis was performed where there were missing data points (e.g., if an animal did not choose the water option for the whole session). Multiple comparisons using Sidak's *post hoc* test were used when applicable. For analysis of immobility, two-way, repeated measures ANOVA was used, with sex and rFSS block as factors or surgical condition and block as factors. All statistical analysis was carried out using Prism 9 (GraphPad).

## Results

**Figure 2:**
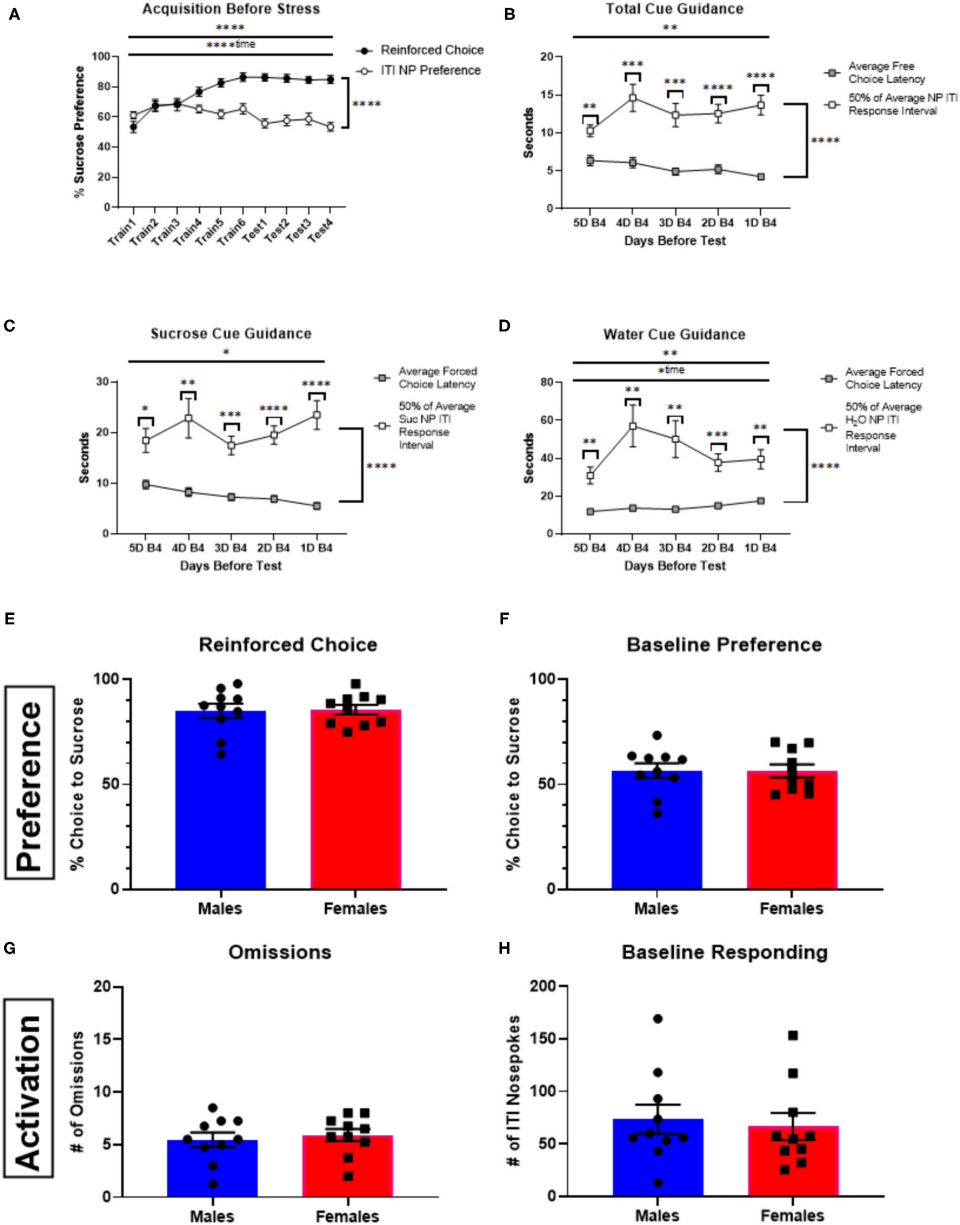
Acquisition behavior, cue-guidance verification, and sex comparisons of behavior in a stress-naïve state. **(A)** Percent sucrose receptacle preference: reinforced choice percentage vs. baseline preference represented by nosepokes made during the ITI (±SEM) up to the day of stress (or no stress) for animals not receiving drug manipulation (*n* = 20). Criteria for advancement to test days was for each animal to reach >75% preference for sucrose during free-choice trials. **(B)** Total cue-guidance verification represented by comparing the average free-choice latency (±SEM) to choose to 50% of the average nosepoke ITI response interval (±SEM) in the days leading up to the first test day. **(C)** Cue guidance verification of responses made for the discrete cues associated with sucrose forced choices. **(D)** Cue guidance verification of responses made for the discrete cues associated with water forced choices. **(E–H)** Comparison between male (*n* = 10) and female (*n* = 10) mice for reinforced choice **(E)**, baseline preference **(F)**, number of trial omissions **(G)**, and baseline responding **(H)**. Error bars are (±SEM). *P < 0.05, **P < 0.01, ***P < 0.001, ****P < 0.0001.

During training, mice acquired a preference for sucrose, as ascertained from free-choice trials (Figure 2A). They also acquired a preference for the sucrose-associated nose-poke port when making exploratory (non-reinforced) nose pokes during the intertrial interval (Figure 2A). The rate of nose-poke responses during the intertrial interval between the 48 trials during the training phase was once every 25.57 +/−9.66 s. If responding during sucrose or water cue presentation (i.e., during a forced- or free-choice trial) was simply baseline responding, then we would anticipate the average latency to respond following cue presentation be half of this time. However, this latency was significantly shorter (Figures 2B–D) for free-choice responses [*F*_(1.38)_ = 42.39, *p* < 0.0001] with a significant interaction [*F*_(4, 152)_ = 3.803, *p* = 0.0056] indicating that mice were engaging to the cue presentation. The same was true for force-choice trials: latencies were significantly shorter than half of the average time between responses during the ITI for their respective receptacle [sucrose: *F*_(1, 38)_ = 45.79, *p* < 0.0001; interaction: *F*_(4, 152)_ = 2.66, *p* = 0.034; water: *F*_(1, 38)_ = 25.70, *p* < 0.0001; interaction: *F*_(4, 152)_ = 3.59, *p* = 0.0081].

Sex differences were not observed when assessing behavior with the primary performance metrics (reinforced choice, *p* = 0.8825; baseline preference, *p* = 0.9674; omissions, *p* = 0.6445; baseline responding, *p* = 0.7173; Figures 2E–H). In fact, the only significant sex difference observed in task performance was that males had longer free-choice latencies than females for both sucrose (*p* < 0.05) and water (*p* < 0.01), but not forced-choice trial latencies (see Supplementary Figure 2).

**Figure 3:**
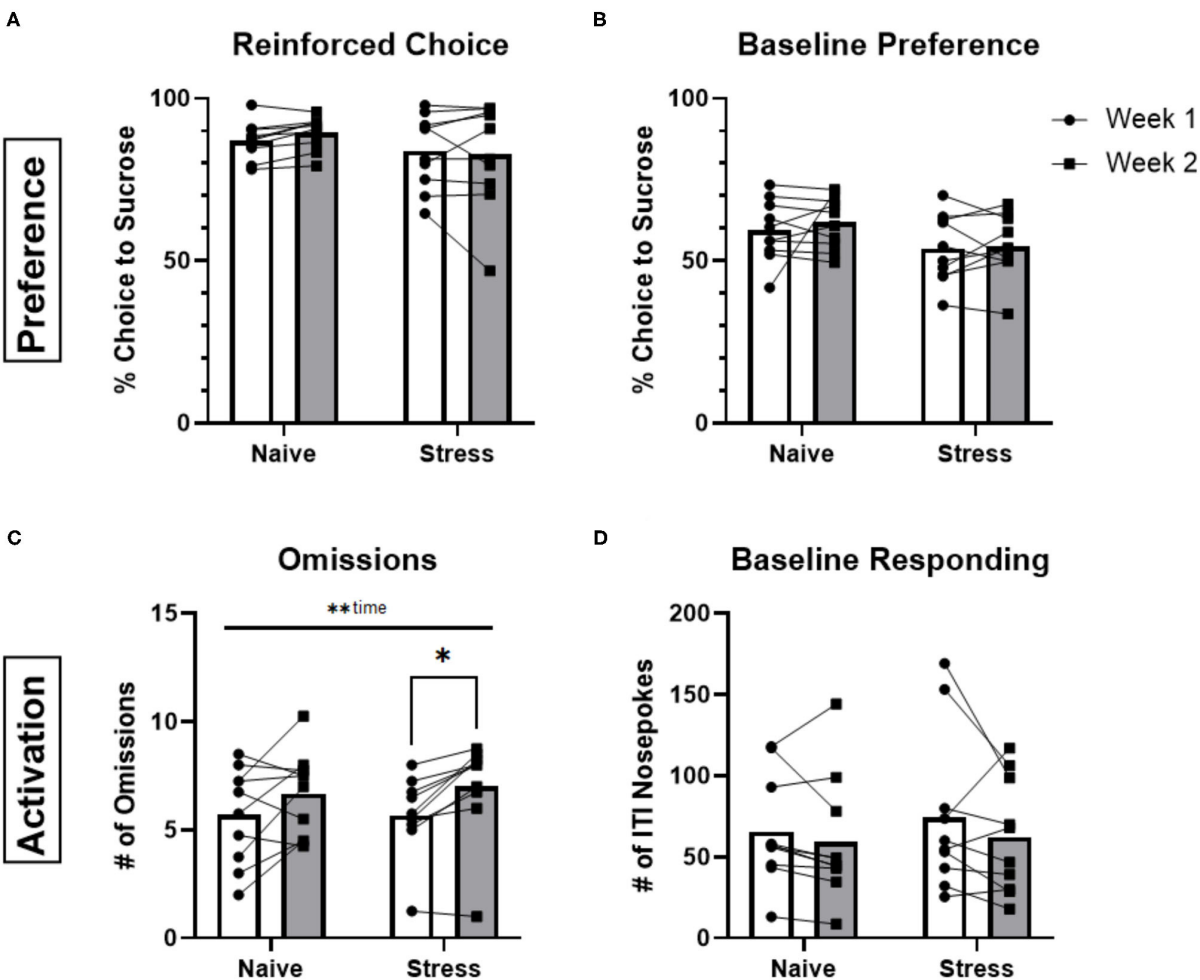
Stress vs. stress-naïve behaviors. **(A)** Averaged percentage of reinforced choice for the sucrose liquid receptacle in free-choice trials for animals in sessions before (week 1) and after (week 2) the rFSS (*n* = 10) or 2 rest days (*n* = 10). **(B)** Percentage of ITI nosepokes made during the session for the sucrose liquid receptacle compared to water receptacle nosepokes during the ITI. **(C)** Total number of omissions made during the sessions. **(D)** Total number of ITI nosepokes made at all during the sessions. Error bars are (±SEM). *P < 0.05, **P < 0.01.

We next tested whether performance was sensitive to stress exposure. Following one testing period (Week 1) animals underwent repeated forced-swim stress and were retested during a second period (Week 2). Stress-naïve control animals were tested during weeks 1 and 2 but were not exposed to swim stress in the interim. Reinforced choice, baseline preference and baseline responding were not significantly different between weeks 1 and 2 for either control or stress groups (*p* > 0.05; Figures 3A,B,D). There was a significant main effect of time for omissions [*F*_(1,18)_ = 13.92, *p* = 0.0015] and this metric was significantly increased between weeks 1 and 2 for the stress group (*p* = 0.0133; Figure 3C). However, there was no stress-by-time interaction [*F*_(1,18)_ = 0.3621, *p* = 0.5549], suggesting that this result was not an effect of stress but driven by a general increase in omissions over time. Likewise, there were no detectable differences between the control or stressed groups for either free- and forced-choice latencies (see Supplementary Figure 3). Furthermore, there were no significant effects (*p* > 0.05) of stress across sexes as indicated by the lack of significant interactions pertaining to sex (sex x stress, sex x time, sex x stress x time) when analyzed by three-way ANOVA with sex, time, and stress as factors (data not shown).

While there were no apparent effects of stress on the task performance, we know that this same stressor exposure robustly changes the ability for CRF to increase dopamine (12, 26, 27). Therefore, we tested the roles of dopamine and CRF on the decision-making task before and after stress.

**Figure 4:**
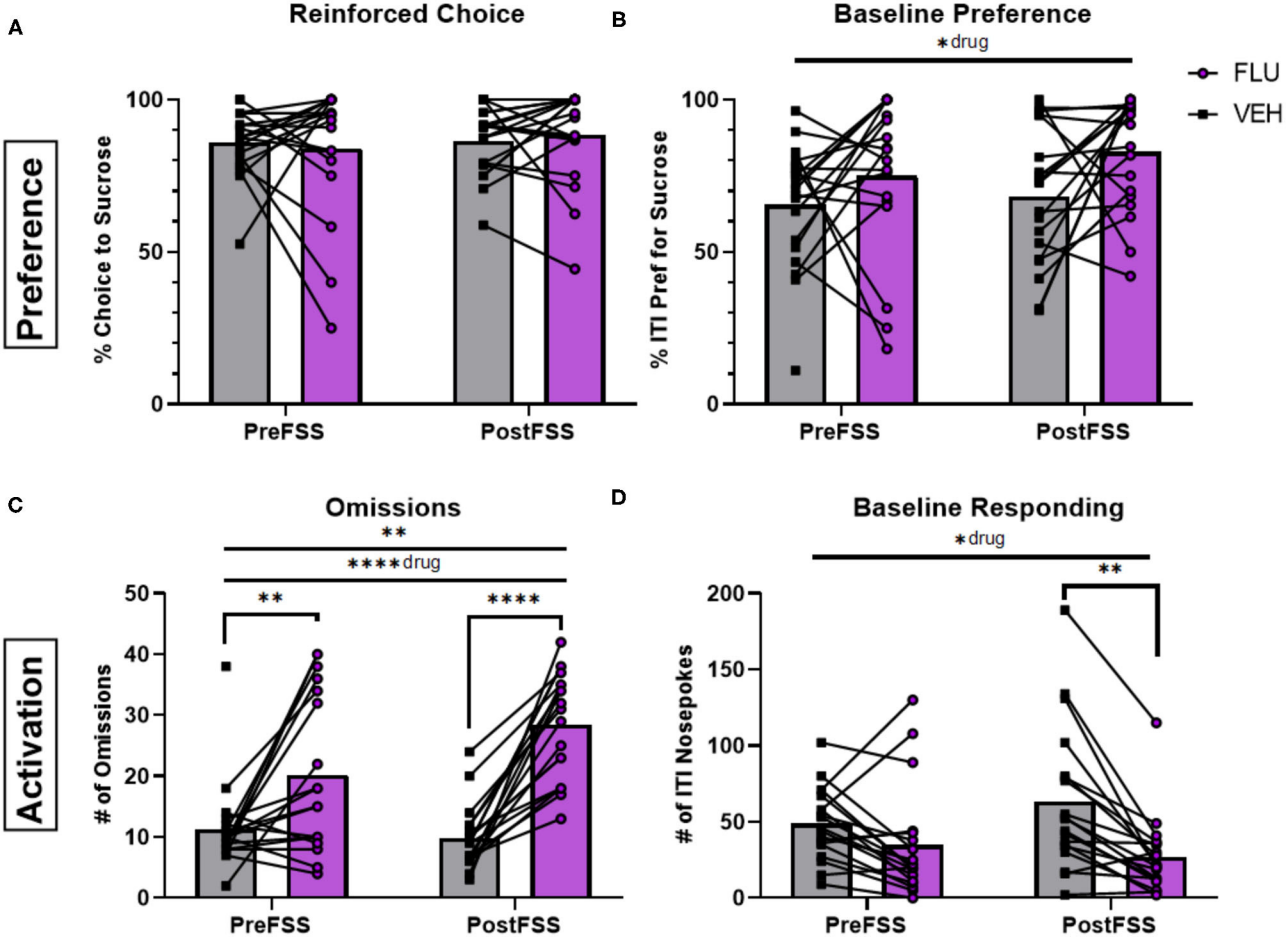
Influence of dopamine antagonism on decision-making behaviors. **(A)** Averaged percentage of reinforced choice for the sucrose liquid receptacle in free-choice trials in sessions before (PreFSS) and after (PostFSS) the rFSS for animals receiving either flupenthixol (20 μg/0.5 μL; *n* = 19) or vehicle (physiological saline; *n* = 19). **(B)** Percentage of ITI nosepokes made during the session for the sucrose liquid receptacle compared to water receptacle nosepokes during the ITI. **(C)** Total number of omissions made during the sessions. **(D)** Total number of ITI nosepokes made at all during the sessions. Error bars are (±SEM). *P < 0.05, **P < 0.01, ****P < 0.0001.

Administration of the dopamine-receptor antagonist, flupenthixol (20 μg in 500 nl), into the NAc had modest effects on preference. It increased baseline preference compared to vehicle administration [*F*_(1, 36)_ = 5.544, *p* = 0.0241; Figure 4B] but did not significantly change reinforced choice (*p* > 0.05) (Figure 4A). However, this treatment robustly affected engagement metrics. Flupenthixol increased the number of omissions with a main effect of drug [*F*_(1, 36)_ = 34.39, *p* < 0.0001] and an interaction between drug and time [*F*_(1, 35)_ =8.031, *p* = 0.0076], yielding more robust effects following stress (*p* < 0.001) compared to pre-stress conditions (*p* = 0.0059; Figure 4C). Baseline responding was decreased by flupenthixol with a main effect of drug [*F*_(1, 36)_ = 7.172, *p* = 0.0111] and the effect was stronger following stress (*p* = 0.0031; Figure 4D), with a significant interaction between drug and stress [*F*_(1, 35)_ = 7.198, *p* = 0.0111]. Similarly, flupenthixol significantly increased free-choice latencies of both sucrose [*F*_(1, 36)_ = 8,629, *p* = 0.0057] and water [*F*_(1, 31)_ = 9.469, *p* = 0.0043] choices, with a stronger effect following stress for both (*p* < 0.01). Forced-choice trials were affected by drug treatment for sucrose, but not water, trials [*F*_(1, 36)_ = 7.946, *p* = 0.0078] an effect driven by stress (*p* < 0.05; see in Supplementary Figure 4).

Once again, these effects were not sexually dimorphic as indicated by the lack of interaction (*p* > 0.05) pertaining to sex (sex x drug, sex x time, sex x drug x time) when analyzed by three-way ANOVA with sex, time, and drug as factors (data not shown).

**Figure 5:**
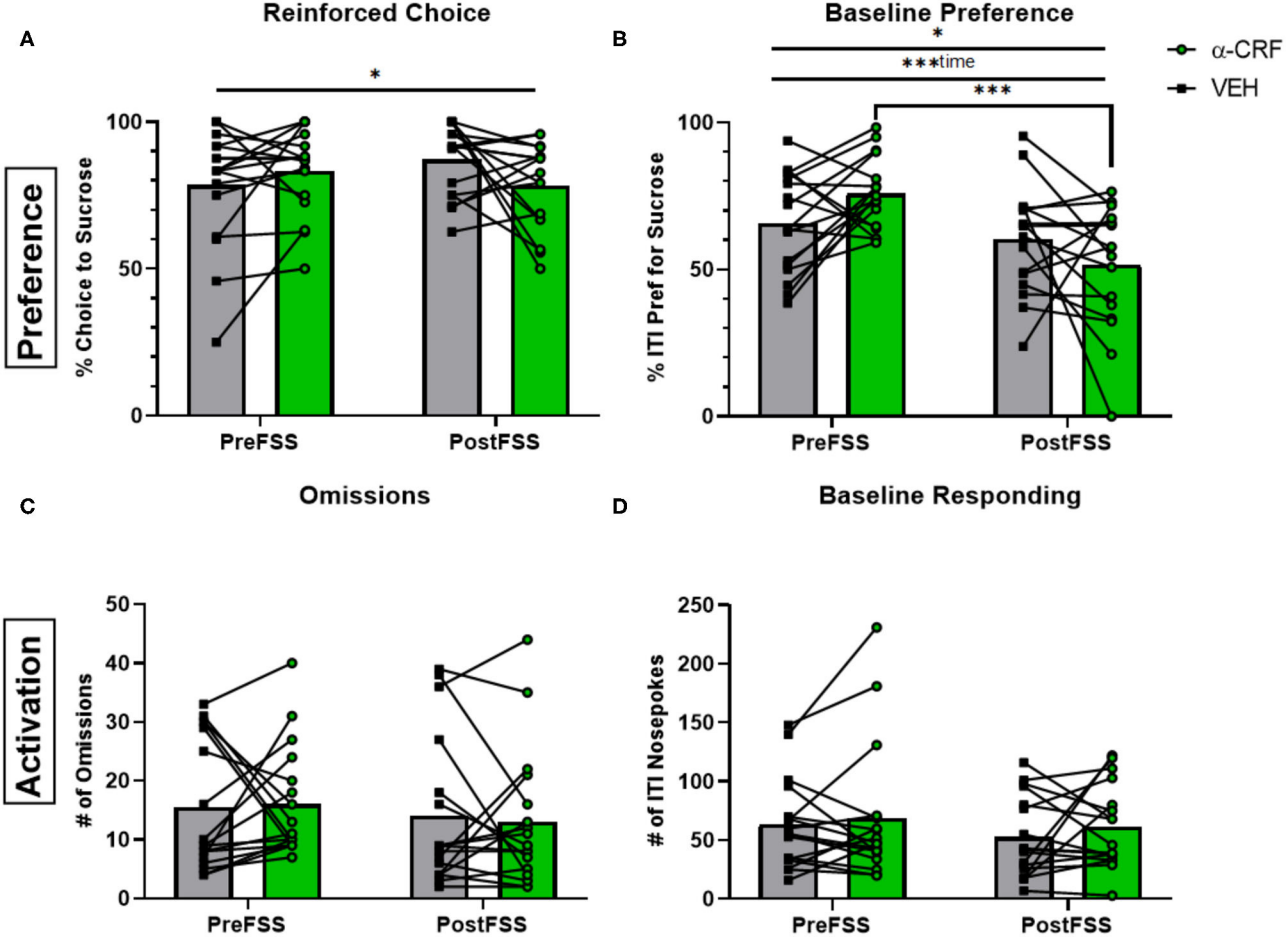
Influence of CRF antagonism on decision-making behaviors. **(A)** Averaged percentage of reinforced choice for the sucrose liquid receptacle in free-choice trials in sessions before (PreFSS) and after (PostFSS) the rFSS for animals receiving either α-helical CRF_(9−41)_ (500 ng/200 nL; Tocris Bioscience; *n* = 17) or its vehicle (0.01% acetic acid in lactated ringer's solution; *n* = 17). **(B)** Percentage of ITI nosepokes made during the session for the sucrose liquid receptacle compared to water receptacle nosepokes during the ITI. **(C)** Total number of omissions made during the sessions. **(D)** Total number of ITI nosepokes made at all during the sessions. Error bars are (±SEM). *P < 0.05, ***P < 0.001.

In contrast to the effects of flupenthixol, administration of the CRF antagonist, α-helical CRF (500 ng in 200 nl), affected preference rather than engagement. There was a significant interaction between stress and drug for both reinforced choice [*F*_(1, 32)_ = 4.447, *p* = 0.0429] and baseline preference [*F*_(1, 32)_ = 6.116, *p* = 0.0189; Figures 5A,B]. Interestingly, there was a main effect of time [before and after stress; *F*_(1, 32)_ = 15.17, *p* < 0.001] where α-helical CRF administration following stress elicited a reduced preference compared to microinjection during the stress naïve state (*p* < 0.001). However, neither of the engagement metrics were significantly affected by CRF antagonism (Figures 5C,D).

When analyzing additional performance metrics, only the water free-choice latency had an interaction between drug and stress [*F*_(1,24)_ = 7.236, *p* = 0.0128] where drug administration after stress led to a higher latency compared to before-stress conditions (*p* = 0.0138). None of the other latency types had an effect from either stress or drug (see in Supplementary Figure 5).

These effect of α-helical CRF_(9−41)_ did not differ between sexes as indicated by the lack of interaction (*p* > 0.05) pertaining to sex (sex x drug, sex x time, sex x drug x time) when analyzed by three-way ANOVA with sex, time, and drug as factors (data not shown).

## Discussion

In the current work, mice performed an operant task to make concurrent choices between qualitatively different outcomes: water and sucrose. They exhibited preferences for sucrose when executing choices, and for the manipulandum associated with sucrose, during the intervals when choices were unavailable. However, when these animals were exposed to stress, robust changes in task performance were not observed in the subsequent sessions. Previously, stress effects have been demonstrated on choices between rewards available for different response requirements (28). Nonetheless, in the same study, an effect of stress was not observed on choices between *quantitatively* different rewards when they were available for equal response costs. Likewise, in the current work, we did not observe stress effects on choices between *qualitatively* rewards available for equal responses costs. While this result is not unprecedented, it is still surprising given that the stress procedure we utilized produces enduring changes in CRF regulation of mesolimbic dopamine signaling (12, 26, 27). Even though the observed behavior metrics did not appear to be altered by stress, we did find evidence that the neurochemical regulation of the behavior had changed as indicated by treatment by stress interactions using CRF or dopamine antagonists.

Based on our previous results, we had hypothesized that CRF and dopamine act in series to regulate appetitive behavior. However, contrary to this notion, the transmitter systems regulated different components of the behavior. Although the effects of CRF antagonism were subtle, they exclusively impacted choice metrics, without effect on activation. In contrast, dopamine antagonism primarily regulated activations metrics, with minimal effects on choice.

While it comes as no surprise that dopamine affects activation and engagement in the task, many theories also place a significant role for dopamine in choice. Indeed, there are certainly downstream means by which mesolimbic dopamine may influence choice (e.g., through learning mechanisms), but several reports indicate that dopamine does not have immediate effects on preference between concurrent choices in several types of decision-making tasks. For example, dopamine antagonists reduce the ability of a Pavlovian cue to invigorate instrumental behavior without affecting the ability of the cue to drive preference between concurrent options (29). Similarly, mice, that are genetically rendered so their dopamine-containing neurons are deficient of dopamine, have a dramatic reduction in their liquid intake when they are allowed to choose between freely available water and sucrose, but the relative proportion of the two reinforcers that they consume is not significantly different from controls (30). However, this is not the full story, as dopamine's role is more nuanced and can influence certain types of choices. Most notably, preferences between outcomes of different magnitudes, available for different amounts of work (31, 32) or different delays before their delivery (33) are both altered by dopamine antagonists. Consistent with this nuanced role, dopamine transmission does not encode all economic parameters commensurate with the degree they influence choice (34, 35). In fact, this observation can be exploited to create conditions where animals consistently select an option that elicits the least dopamine release of the available options (36). Nonetheless, the current results where dopamine antagonism in NAc reduced invigoration but not preference between sucrose and water is very consistent with results from the aforementioned global genetic reduction of dopamine on sucrose and water choices observed by Cannon and Palmiter (30).

The dose of flupenthixol was chosen to produce effects in the NAc without the gross motor impairment associated with dopamine receptor antagonism throughout the striatum. Importantly, it is difficult, if not impossible, to completely parse motivated behavior and locomotion in an operant task. As such, the overall reduction in responding during the ITI may be indicative of reduced exploration (motivated behavior) or locomotion. In either case, this effect was more robust on the water receptacle than the sucrose receptacle, leading to an increased “baseline preference” even though preference was unaffected during reinforced choices. While this result is open to interpretation, it is consistent with reduced overall locomotion within the chamber with less traversing between receptacles.

With regard to CRF antagonism, there have also been mixed outcomes in the aspects of task parameters affected (13, 18, 37). Therefore, for the purpose of the current work to ascertain the cooperability of dopamine and CRF signaling, it was important that dopamine and CRF antagonists were compared in the same task. In doing so, we established a disassociation between their effects on task parameters, demonstrating that the two systems are acting in parallel, not serially, in the regulation of the behavior.

It should be noted that for each antagonist a single dose was used in this study. Those doses were based on previous studies where infusions into the NAc produced specific effects on behavior without gross motor impairment. Additional doses were not included because the use of multiple doses of locally infused drugs is complicated by changes in the sphere of influence. That is, the drug diffuses from the injection site without reaching steady state and so higher concentrations impact more tissue, potentially outside the region of interest, and lower concentrations impact a smaller region. Therefore, to achieve site-specific targeting, one loses the precision of a concentration-response profile.

The rFSS (20, 21, 38) used in this study was used to probe sex-specific effects. It has been shown that rFSS could lead to long-lasting alterations in how dopamine and CRF interact within the nucleus accumbens or reward-related area of female mice (27, 39). However, unlike reports of sex differences in CRF signaling and stress-responsivity (40–46), we found no sexually dimorphic effects in our task of stress or receptor antagonism. In fact, the only sex difference we observed was free-choice latencies where stress-naïve males took longer to make choices than their female counterparts (see Supplementary Figures 2A,B).

In summary, the current work tested the cooperability in CRF and dopamine signaling in the NAc during concurrent choices between qualitatively different rewards under different stress-exposure histories in male and female subjects. We found no sex differences in the regulation of the decision-making task by mesolimbic dopamine or CRF and, contrary to our working hypothesis, CRF and dopamine act independently during this decision-making task, regulating distinct aspects of the behavior.

## Data Availability Statement

The original contributions presented in the study are included in the article/Supplementary Material, further inquiries can be directed to the corresponding author/s.

## Ethics Statement

The animal study was reviewed and approved by University of Washington Institutional Animal Care and Use Committee.

## Author Contributions

RW and PP designed the experiments and wrote the manuscript. RW and KL carried out the experiments. RW analyzed the data. All authors contributed to the article and approved the submitted version.

## Funding

This research was supported by an NSF Graduate Research Fellowship to RW, and NIH grants P50-MH106428 (5877), R01- DA039687, and R37-DA051686 to PP.

## Conflict of Interest

The authors declare that the research was conducted in the absence of any commercial or financial relationships that could be construed as a potential conflict of interest.

## Publisher's Note

All claims expressed in this article are solely those of the authors and do not necessarily represent those of their affiliated organizations, or those of the publisher, the editors and the reviewers. Any product that may be evaluated in this article, or claim that may be made by its manufacturer, is not guaranteed or endorsed by the publisher.
