# Supplementary material for: The Influence of Stress on Decision-Making: Effects of CRF and Dopamine Antagonism in the Nucleus Accumbens
Source: Front Psychiatry. 2022 Jan 25;12:814218. doi: 10.3389/fpsyt.2021.814218 (PMC8821535; doi:10.3389/fpsyt.2021.814218)
Supplement: Supplementary file 1 [file Data_Sheet_1.pdf]

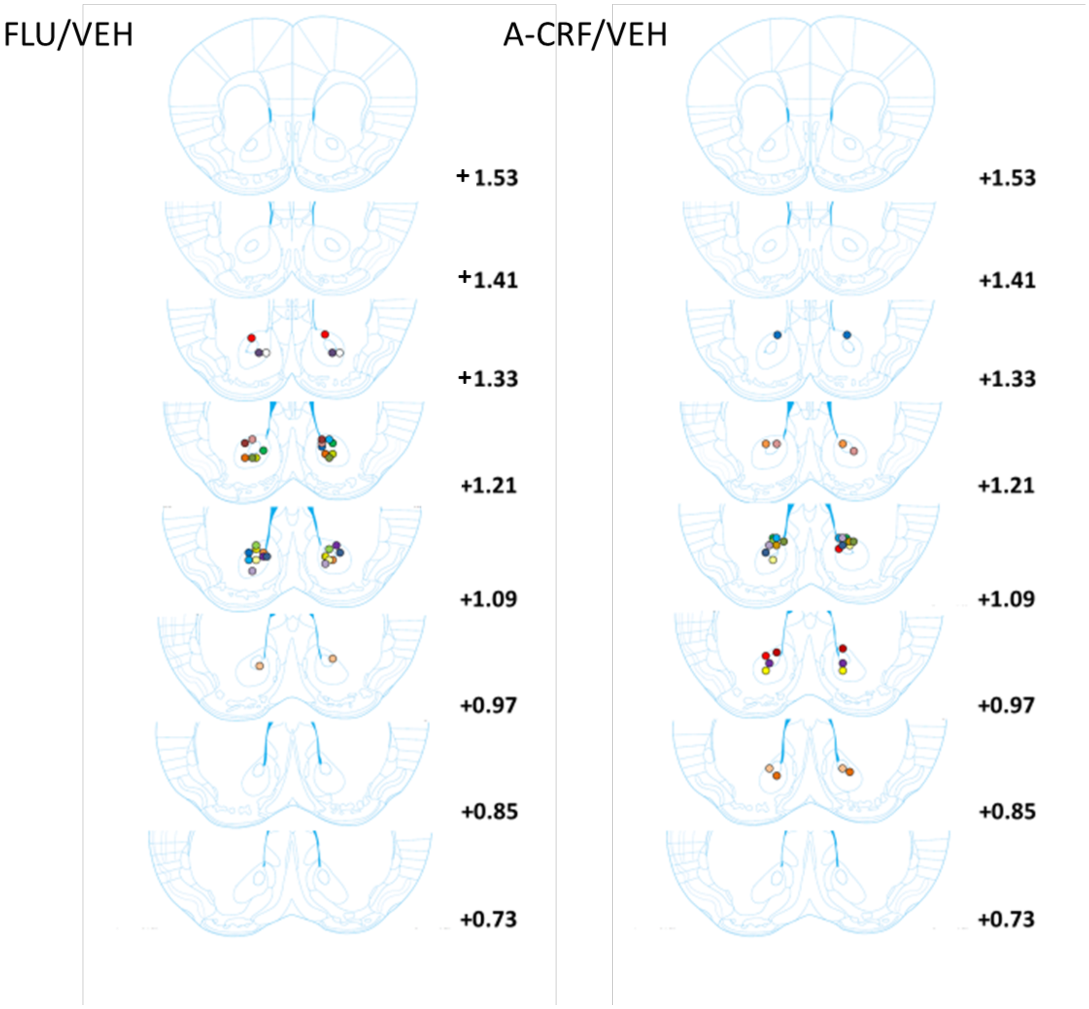

Supplementary Figure 1. Histological verification of injection site. Injection sites for either the cohort that received dopamine antagonism (right) or CRF antagonism (left). Numbers beside each section indicate the anterior distance in mm from bregma. Atlas adapted from Paxinos and Franklin (2008).

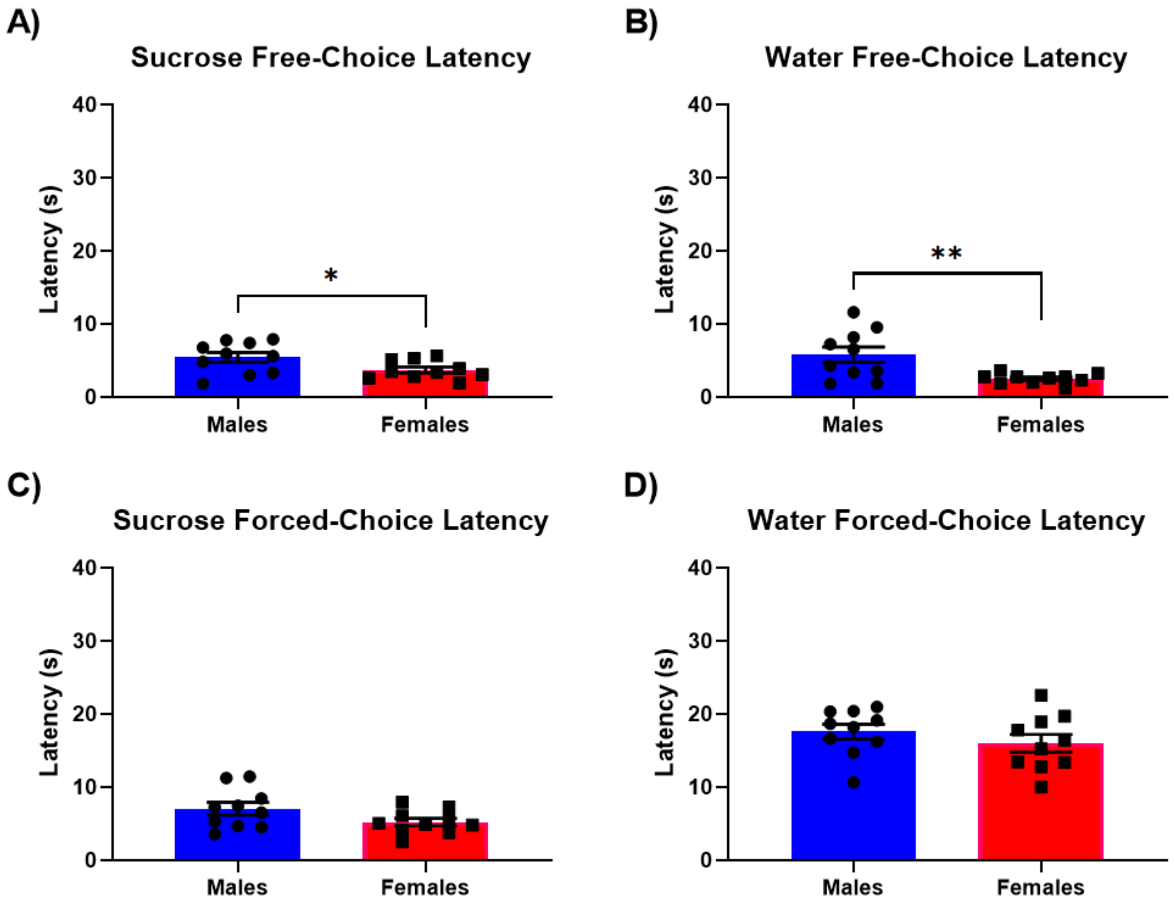

Supplementary Figure 2. Stress-naïve state comparison of male and female latencies to choose. The data here represent the latency from the time of cue onset to the time the animal made a choice during each type of trial for either male (n = 10) or female (n = 10) mice. **A and B)** Male mice in a stress-naïve state seemed to deliberate longer during free-choice trials for either water or sucrose. **C and D)** Males and females did not differ in the latency to respond during forced-choice trials. Error bars are (±SEM). $*P < 0.05$, $**P < 0.01$

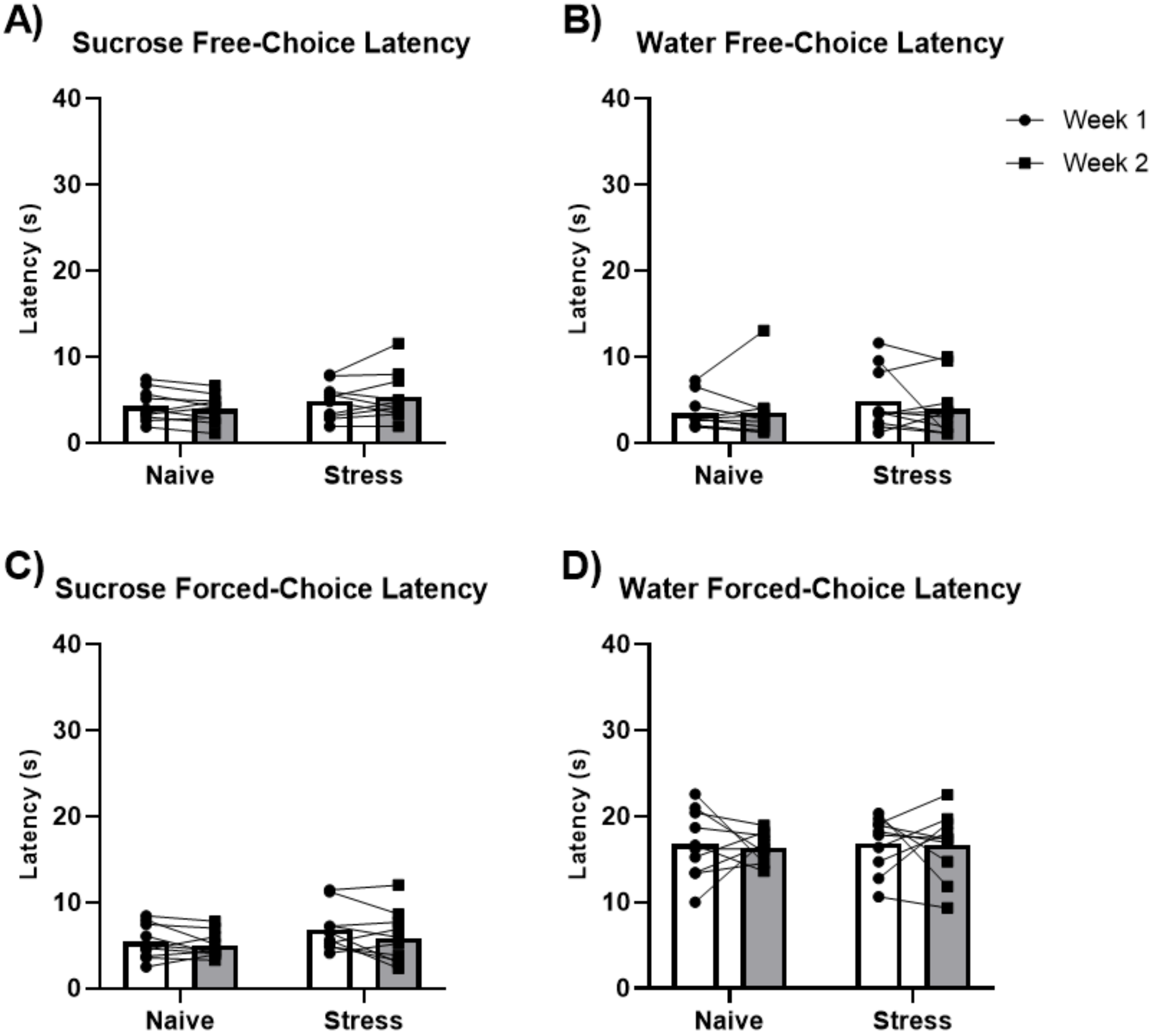

Supplementary Figure 3. Influence of stress on latencies during free- and forced-choice trials. The data here represent the latency from the time of cue onset to the time the animal made a choice during each type of trial for either stressed (n = 10) or stress-naive (n = 10) mice. **A and B)** Stress did not impact latencies to choose during free-choice trials for either water or sucrose. **C and D)** Stress did not impact latencies to respond during forced-choice trials. Week 1 represents the average latencies before the rFSS (or rest days), and Week 2 is the average of latencies following stress manipulation. Error bars are (±SEM).

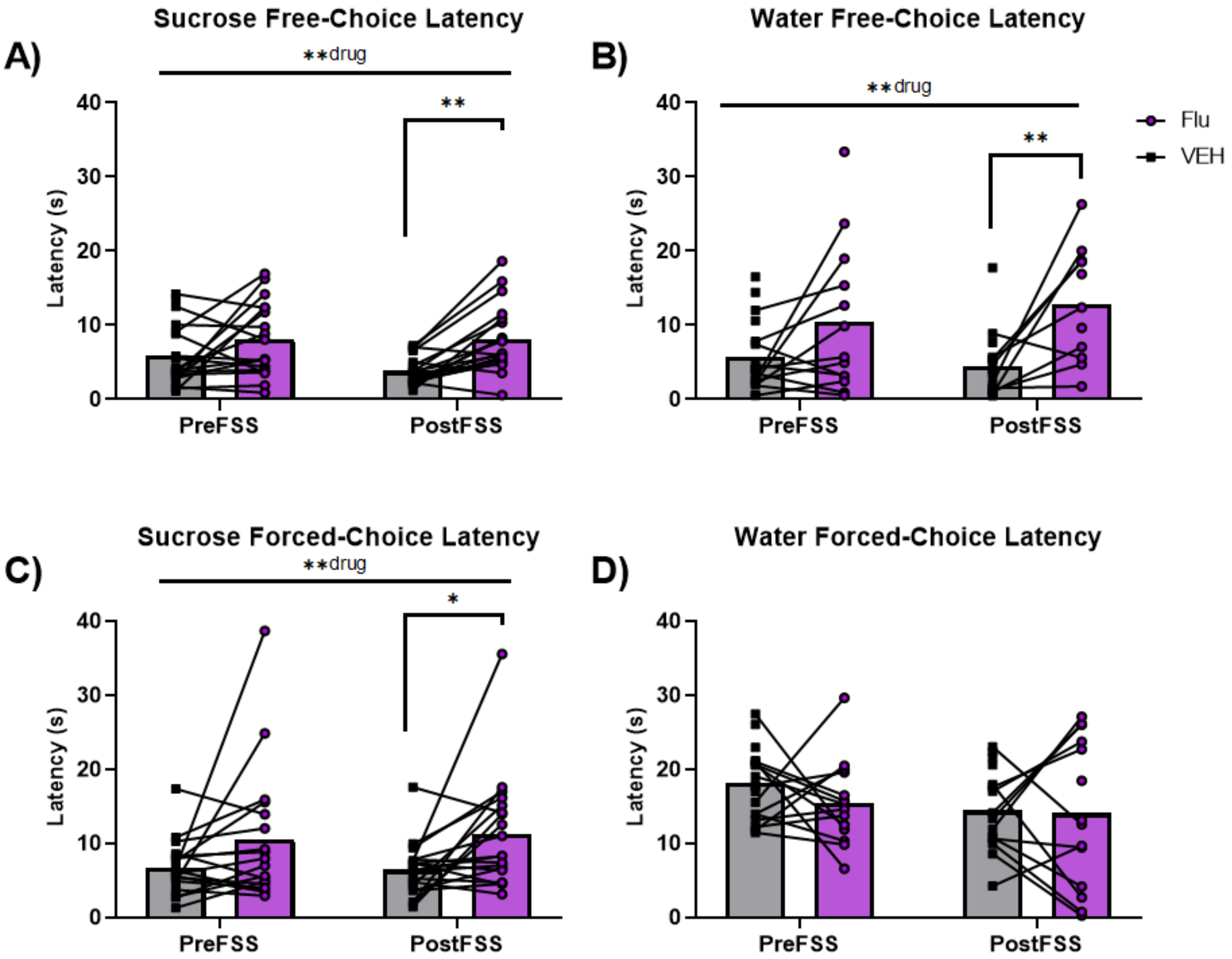

Supplementary Figure 4. Influence of dopamine antagonism on free- or forced-choice latencies. A and B) Flupenthixol (20 µg/0.5 µL; n = 19) or vehicle (physiological saline; n = 19) administered animals differed in their latencies to choose for sucrose and water during free-choice trials and the increase in latency for animals that received flupenthixol was stronger following stress (postFSS). **C)** dopamine antagonism increased the latency to respond during sucrose forced choice trials, especially following stress. **D)** Latencies were not impacted by drug administration during water-forced choice trials. Error bars are (±SEM). *$P < 0.05$, **$P < 0.01$

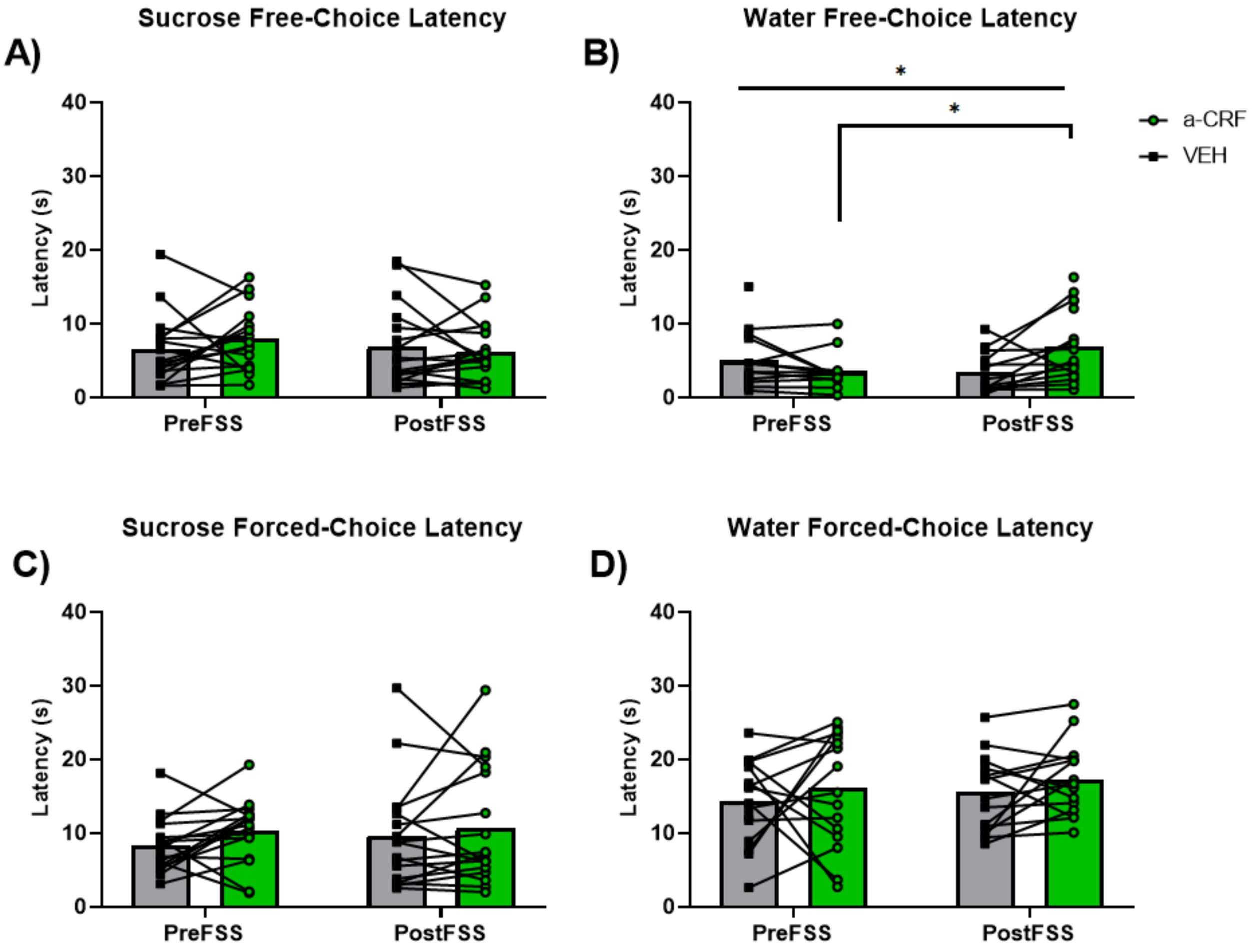

Supplementary Figure 5. Influence of CRF antagonism on free- and forced-choice latencies. A) Animals receiving either α-helical $CRF_{(9\text{-}41)}$ (500 ng/200 nL; Tocris Bioscience; n = 17) or its vehicle (0.01 % acetic acid in lactated ringer's solution; n = 17) did not differ in their latency to choose during free-choice trials. **B)** There existed an overall stress x drug interaction during water free-choice trials and animals that received CRF antagonist after stress had higher latencies than receiving the drug before stress. **C and D)** CRF antagonism did not affect sucrose and water forced-choice latencies to respond to the cue. Error bars are (±SEM). *P < 0.05, **P < 0.01, ***P < 0.001, ****P < 0.0001

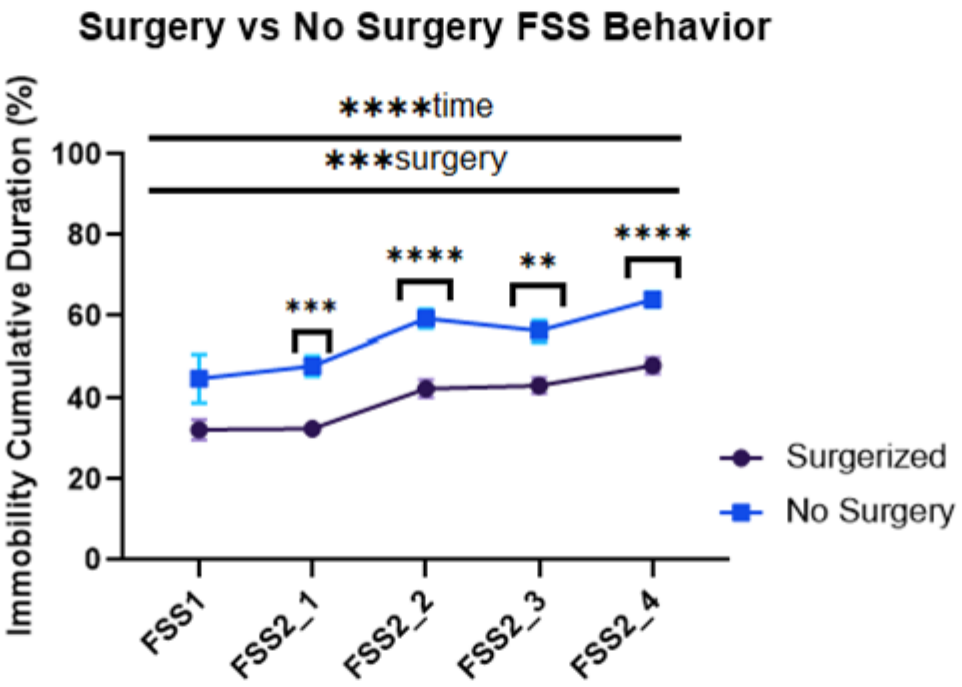

Supplementary Figure 6. Effect of implant surgery on immobility cumulative duration percentages during forced-swim stress. Animals that received bilateral cannulation implants had lower immobility times than animals that did not receive surgery. Data were calculated in percentages to reflect the amount of time spent immobile. FSS1 was 15 minutes. FSS2 were 6-minute bouts separated by 6 minutes. Error bars are (±SEM). *P < 0.05, **P < 0.01, ***P < 0.001, ****P < 0.0001
